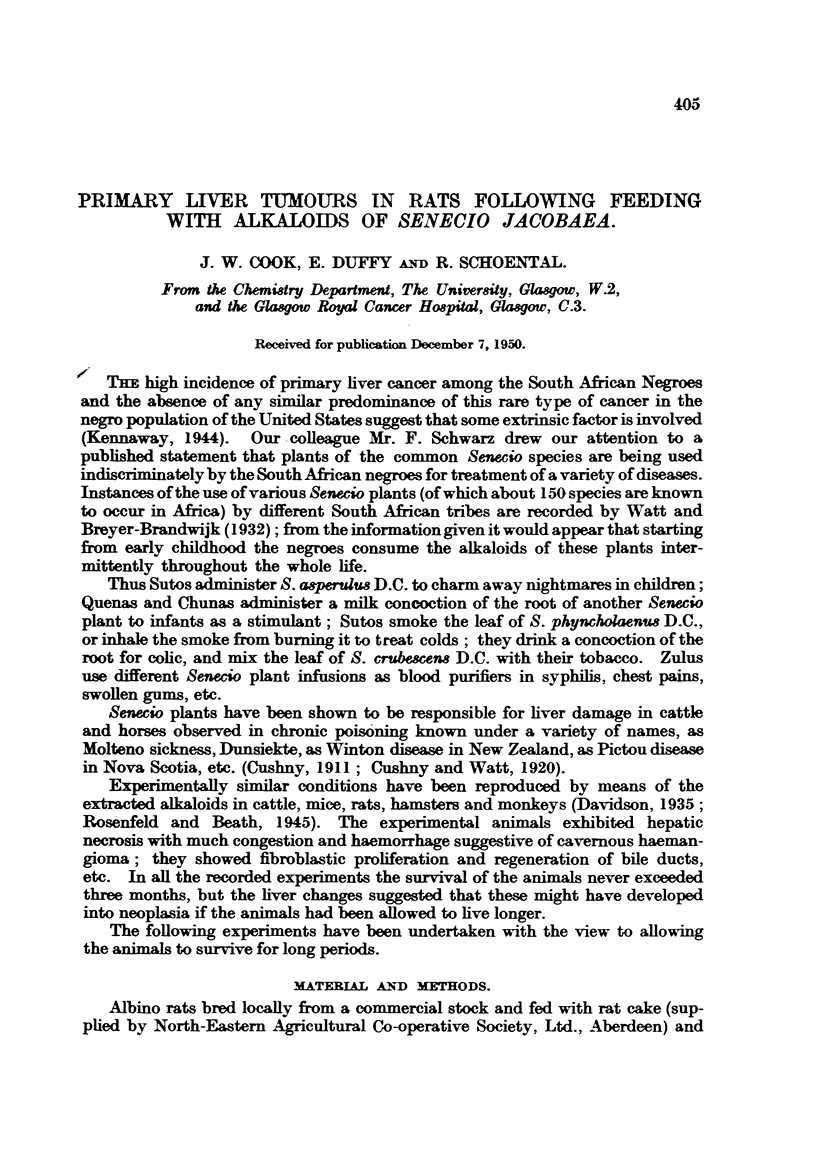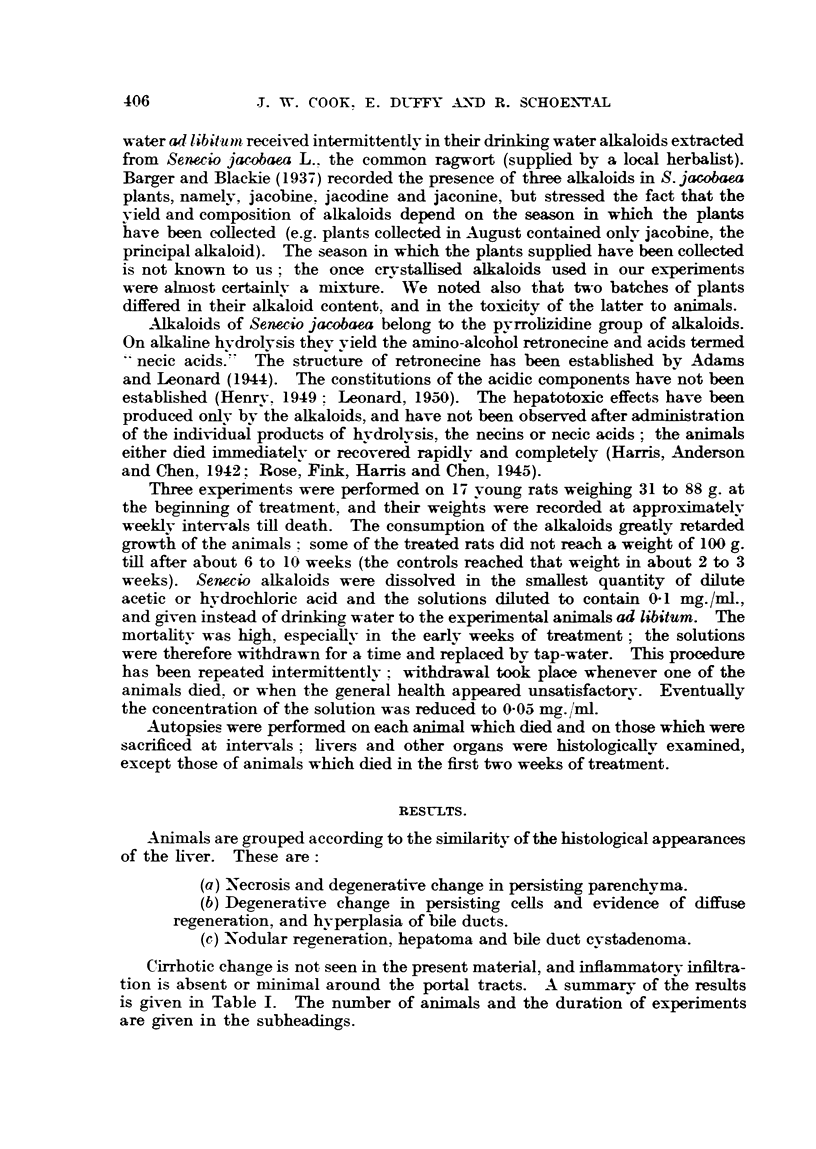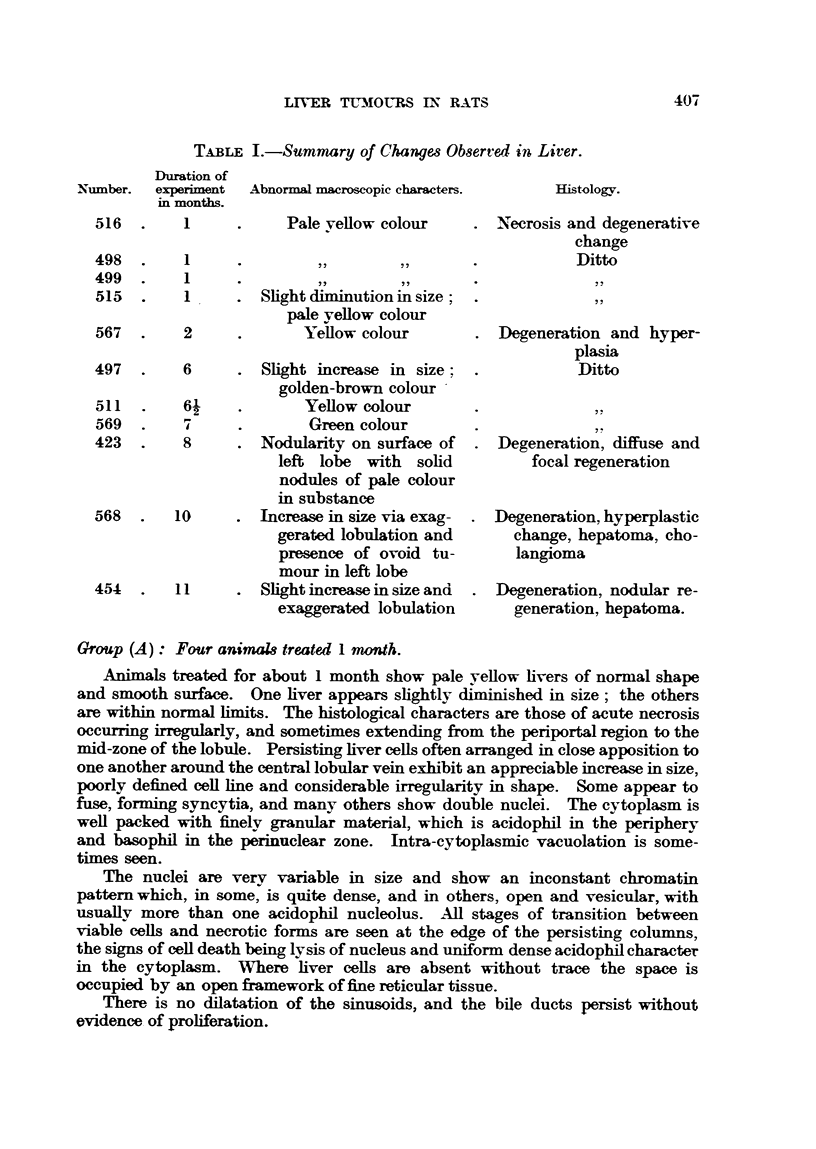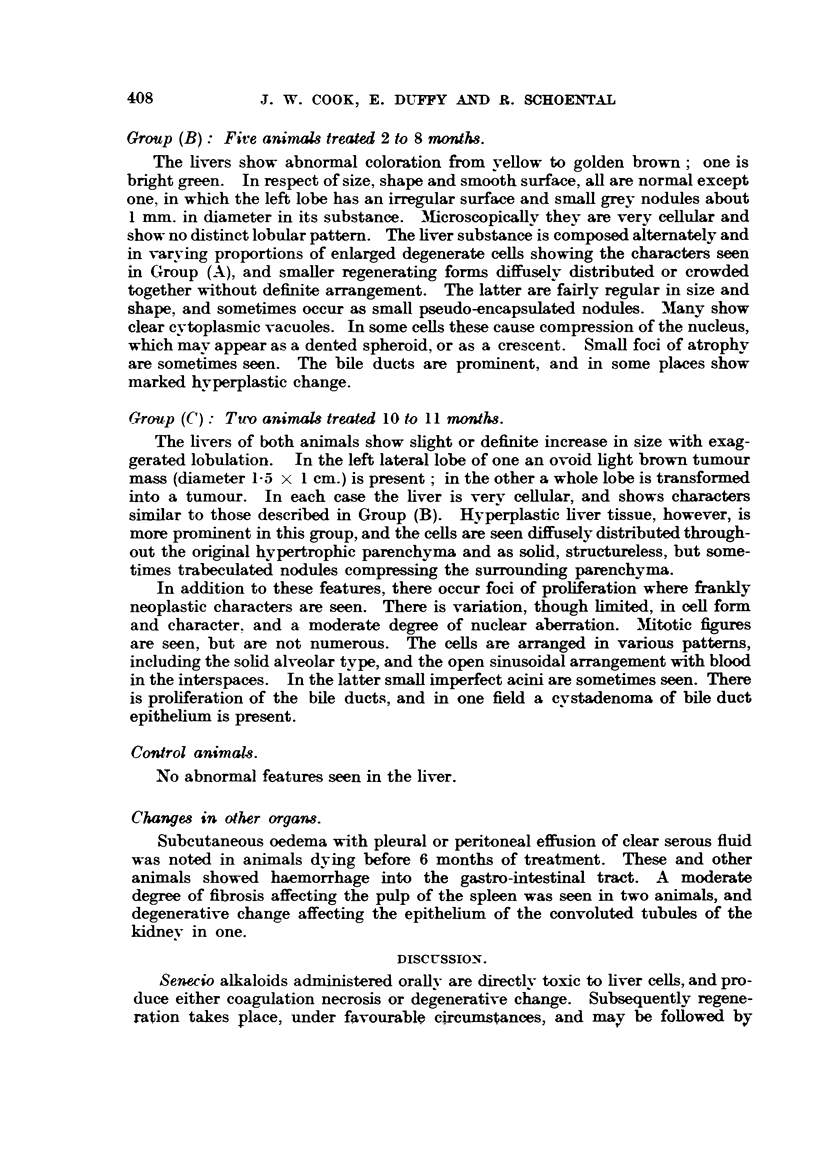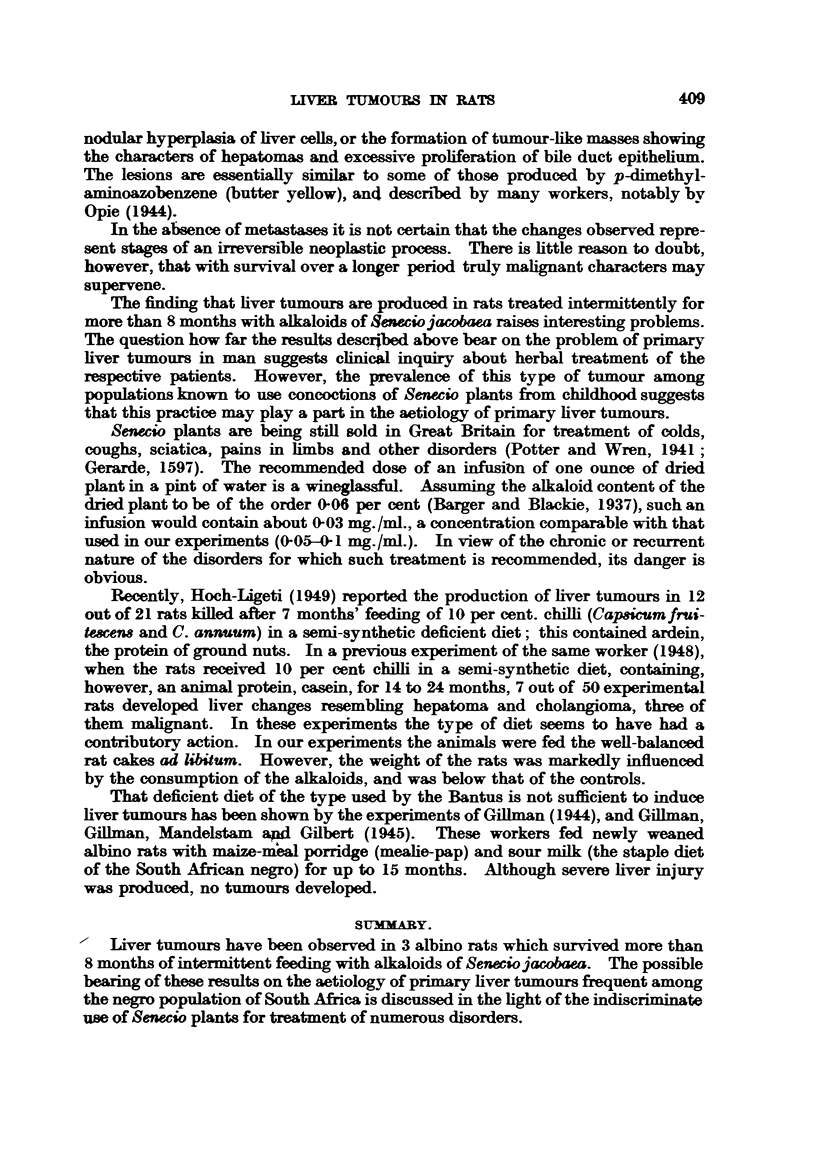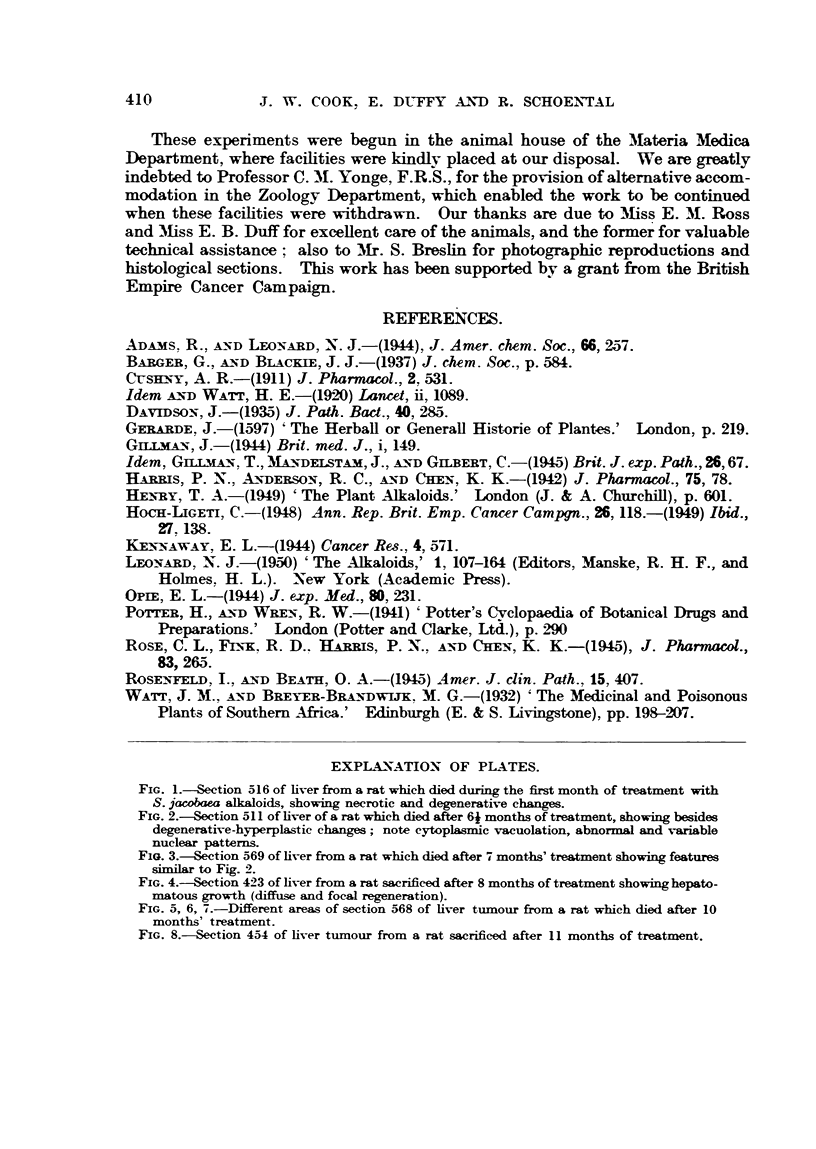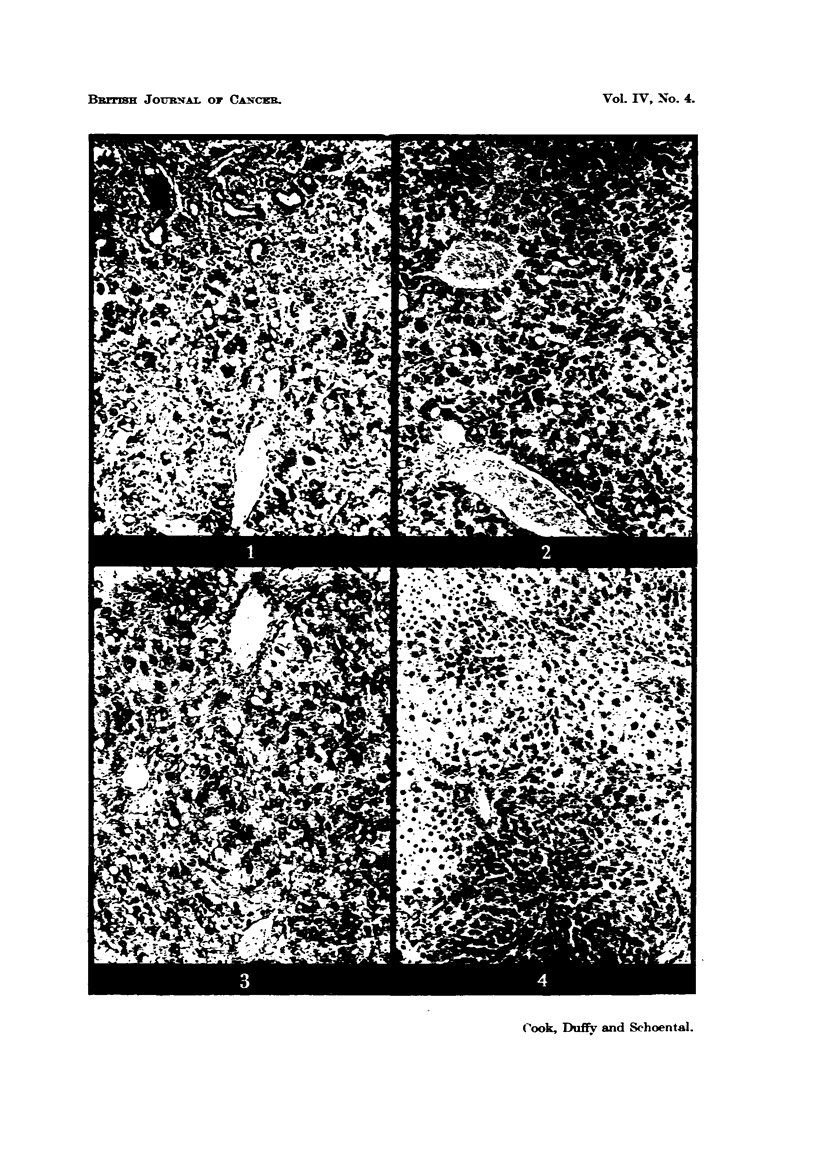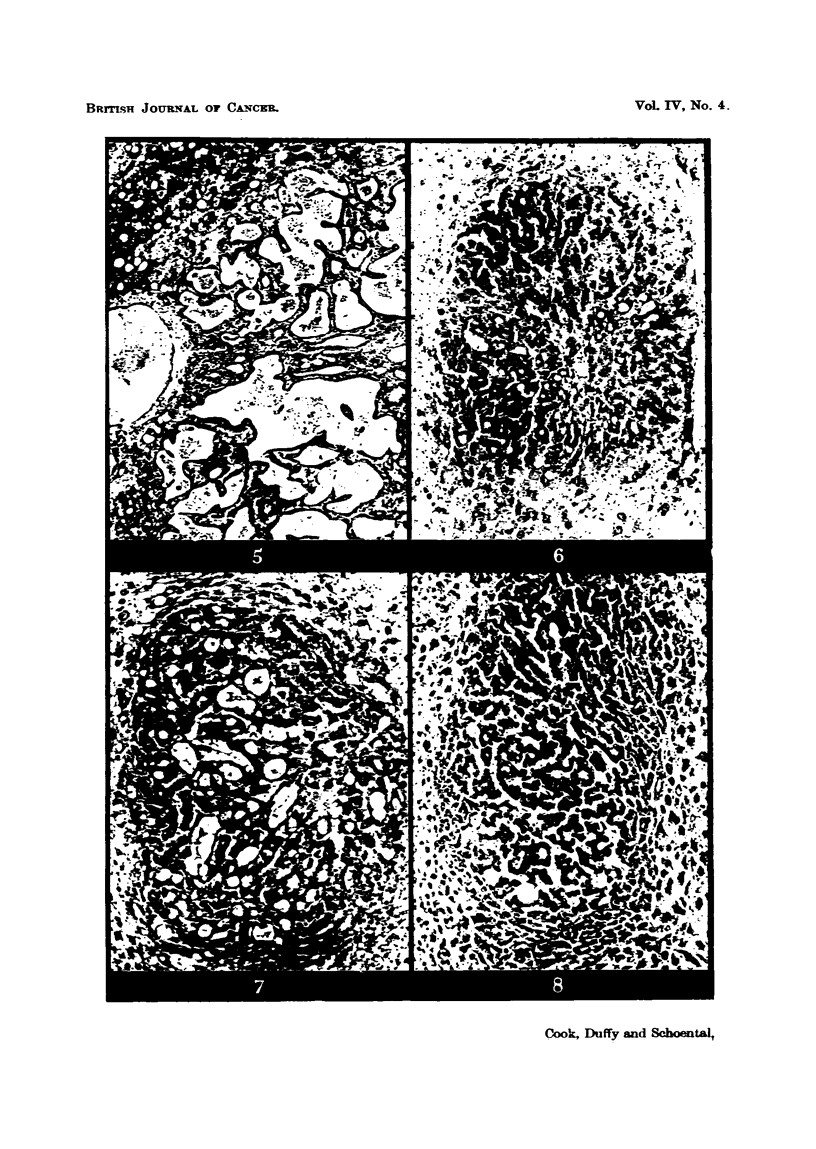# Primary Liver Tumours in Rats following Feeding with Alkaloids of Senecio jacobaea

**DOI:** 10.1038/bjc.1950.39

**Published:** 1950-12

**Authors:** J. W. Cook, E. Duffy, R. Schoental

## Abstract

**Images:**


### PRIMARY LIVER TUMOURS IN RATS FOLLOWING FEEDING WITH ALKALOIDS OF *SENECIO JACOBAEA*.

J. W. COOK, E. DUFFY AND R. SCHOENTAL.

*From the Chemistry Department, The University, Glasgow, W.2, and the Glasgow Royal Cancer Hospital, Glasgow, C.3.*

Received for publication December 7, 1950.

THE high incidence of primary liver cancer among the South African Negroes and the absence of any similar predominance of this rare type of cancer in the negro population of the United States suggest that some extrinsic factor is involved (Kennaway, 1944). Our colleague Mr. F. Schwarz drew our attention to a published statement that plants of the common *Senecio* species are being used indiscriminately by the South African negroes for treatment of a variety of diseases. Instances of the use of various *Senecio* plants (of which about 150 species are known to occur in Africa) by different South African tribes are recorded by Watt and Breyer-Brandwijk (1932); from the information given it would appear that starting from early childhood the negroes consume the alkaloids of these plants intermittently throughout the whole life.

Thus Sutos administer *S. asperulus* D.C. to charm away nightmares in children; Quenas and Chunas administer a milk concoction of the root of another *Senecio* plant to infants as a stimulant; Sutos smoke the leaf of *S. phyncholaenus* D.C., or inhale the smoke from burning it to treat colds; they drink a concoction of the root for colic, and mix the leaf of *S. crubescens* D.C. with their tobacco. Zulus use different *Senecio* plant infusions as blood purifiers in syphilis, chest pains, swollen gums, etc.

*Senecio* plants have been shown to be responsible for liver damage in cattle and horses observed in chronic poisoning known under a variety of names, as Molteno sickness, Dunsiekte, as Winton disease in New Zealand, as Pictou disease in Nova Scotia, etc. (Cushny, 1911; Cushny and Watt, 1920).

Experimentally similar conditions have been reproduced by means of the extracted alkaloids in cattle, mice, rats, hamsters and monkeys (Davidson, 1935; Rosenfeld and Beath, 1945). The experimental animals exhibited hepatic necrosis with much congestion and haemorrhage suggestive of cavernous haemangioma; they showed fibroblastic proliferation and regeneration of bile ducts, etc. In all the recorded experiments the survival of the animals never exceeded three months, but the liver changes suggested that these might have developed into neoplasia if the animals had been allowed to live longer.

The following experiments have been undertaken with the view to allowing the animals to survive for long periods.

### MATERIAL AND METHODS.

Albino rats bred locally from a commercial stock and fed with rat cake (supplied by North-Eastern Agricultural Co-operative Society, Ltd., Aberdeen) and water *ad libitum* received intermittently in their drinking water alkaloids extracted from *Senecio jacobaea* L., the common ragwort (supplied by a local herbalist). Barger and Blackie (1937) recorded the presence of three alkaloids in *S. jacobaea* plants, namely, jacobine, jacodine and jaconine, but stressed the fact that the yield and composition of alkaloids depend on the season in which the plants have been collected (e.g. plants collected in August contained only jacobine, the principal alkaloid). The season in which the plants supplied have been collected is not known to us ; the once crystallised alkaloids used in our experiments were almost certainly a mixture. We noted also that two batches of plants differed in their alkaloid content, and in the toxicity of the latter to animals.

Alkaloids of *Senecio jacobaea* belong to the pyrrolizidine group of alkaloids. On alkaline hydrolysis they yield the amino-alcohol retronecine and acids termed "necic acids." The structure of retronecine has been established by Adams and Leonard (1944). The constitutions of the acidic components have not been established (Henry, 1949 ; Leonard, 1950). The hepatotoxic effects have been produced only by the alkaloids, and have not been observed after administration of the individual products of hydrolysis, the necins or necic acids ; the animals either died immediately or recovered rapidly and completely (Harris, Anderson and Chen, 1942; Rose, Fink, Harris and Chen, 1945).

Three experiments were performed on 17 young rats weighing 31 to 88 g. at the beginning of treatment, and their weights were recorded at approximately weekly intervals till death. The consumption of the alkaloids greatly retarded growth of the animals ; some of the treated rats did not reach a weight of 100 g. till after about 6 to 10 weeks (the controls reached that weight in about 2 to 3 weeks). *Senecio* alkaloids were dissolved in the smallest quantity of dilute acetic or hydrochloric acid and the solutions diluted to contain 0·1 mg./ml., and given instead of drinking water to the experimental animals *ad libitum*. The mortality was high, especially in the early weeks of treatment ; the solutions were therefore withdrawn for a time and replaced by tap-water. This procedure has been repeated intermittently ; withdrawal took place whenever one of the animals died, or when the general health appeared unsatisfactory. Eventually the concentration of the solution was reduced to 0·05 mg./ml.

Autopsies were performed on each animal which died and on those which were sacrificed at intervals ; livers and other organs were histologically examined, except those of animals which died in the first two weeks of treatment.

### RESULTS.

Animals are grouped according to the similarity of the histological appearances of the liver. These are :

(*a*) Necrosis and degenerative change in persisting parenchyma.
(*b*) Degenerative change in persisting cells and evidence of diffuse regeneration, and hyperplasia of bile ducts.
(*c*) Nodular regeneration, hepatoma and bile duct cystadenoma.

Cirrhotic change is not seen in the present material, and inflammatory infiltration is absent or minimal around the portal tracts. A summary of the results is given in Table I. The number of animals and the duration of experiments are given in the subheadings.

TABLE I.—*Summary of Changes Observed in Liver.*

| Number. | Duration of experiment in months. | Abnormal macroscopic characters. | Histology. |
|---|---|---|---|
| 516 | 1 | Pale yellow colour | Necrosis and degenerative change |
| 498 | 1 | ,, ,, | Ditto |
| 499 | 1 | ,, ,, | ,, |
| 515 | 1 | Slight diminution in size ; pale yellow colour | ,, |
| 567 | 2 | Yellow colour | Degeneration and hyperplasia |
| 497 | 6 | Slight increase in size ; golden-brown colour | Ditto |
| 511 | 6½ | Yellow colour | ,, |
| 569 | 7 | Green colour | ,, |
| 423 | 8 | Nodularity on surface of left lobe with solid nodules of pale colour in substance | Degeneration, diffuse and focal regeneration |
| 568 | 10 | Increase in size via exaggerated lobulation and presence of ovoid tumour in left lobe | Degeneration, hyperplastic change, hepatoma, cholangioma |
| 454 | 11 | Slight increase in size and exaggerated lobulation | Degeneration, nodular regeneration, hepatoma. |

*Group (A) : Four animals treated* 1 *month.*

Animals treated for about 1 month show pale yellow livers of normal shape and smooth surface. One liver appears slightly diminished in size ; the others are within normal limits. The histological characters are those of acute necrosis occurring irregularly, and sometimes extending from the periportal region to the mid-zone of the lobule. Persisting liver cells often arranged in close apposition to one another around the central lobular vein exhibit an appreciable increase in size, poorly defined cell line and considerable irregularity in shape. Some appear to fuse, forming syncytia, and many others show double nuclei. The cytoplasm is well packed with finely granular material, which is acidophil in the periphery and basophil in the perinuclear zone. Intra-cytoplasmic vacuolation is sometimes seen.

The nuclei are very variable in size and show an inconstant chromatin pattern which, in some, is quite dense, and in others, open and vesicular, with usually more than one acidophil nucleolus. All stages of transition between viable cells and necrotic forms are seen at the edge of the persisting columns, the signs of cell death being lysis of nucleus and uniform dense acidophil character in the cytoplasm. Where liver cells are absent without trace the space is occupied by an open framework of fine reticular tissue.

There is no dilatation of the sinusoids, and the bile ducts persist without evidence of proliferation.

*Group (B): Five animals treated* 2 *to* 8 *months.*

The livers show abnormal coloration from yellow to golden brown; one is bright green. In respect of size, shape and smooth surface, all are normal except one, in which the left lobe has an irregular surface and small grey nodules about 1 mm. in diameter in its substance. Microscopically they are very cellular and show no distinct lobular pattern. The liver substance is composed alternately and in varying proportions of enlarged degenerate cells showing the characters seen in Group (A), and smaller regenerating forms diffusely distributed or crowded together without definite arrangement. The latter are fairly regular in size and shape, and sometimes occur as small pseudo-encapsulated nodules. Many show clear cytoplasmic vacuoles. In some cells these cause compression of the nucleus, which may appear as a dented spheroid, or as a crescent. Small foci of atrophy are sometimes seen. The bile ducts are prominent, and in some places show marked hyperplastic change.

*Group (C): Two animals treated* 10 *to* 11 *months.*

The livers of both animals show slight or definite increase in size with exaggerated lobulation. In the left lateral lobe of one an ovoid light brown tumour mass (diameter 1·5 × 1 cm.) is present; in the other a whole lobe is transformed into a tumour. In each case the liver is very cellular, and shows characters similar to those described in Group (B). Hyperplastic liver tissue, however, is more prominent in this group, and the cells are seen diffusely distributed throughout the original hypertrophic parenchyma and as solid, structureless, but sometimes trabeculated nodules compressing the surrounding parenchyma.

In addition to these features, there occur foci of proliferation where frankly neoplastic characters are seen. There is variation, though limited, in cell form and character, and a moderate degree of nuclear aberration. Mitotic figures are seen, but are not numerous. The cells are arranged in various patterns, including the solid alveolar type, and the open sinusoidal arrangement with blood in the interspaces. In the latter small imperfect acini are sometimes seen. There is proliferation of the bile ducts, and in one field a cystadenoma of bile duct epithelium is present.

*Control animals.*

No abnormal features seen in the liver.

*Changes in other organs.*

Subcutaneous oedema with pleural or peritoneal effusion of clear serous fluid was noted in animals dying before 6 months of treatment. These and other animals showed haemorrhage into the gastro-intestinal tract. A moderate degree of fibrosis affecting the pulp of the spleen was seen in two animals, and degenerative change affecting the epithelium of the convoluted tubules of the kidney in one.

### DISCUSSION.

*Senecio* alkaloids administered orally are directly toxic to liver cells, and produce either coagulation necrosis or degenerative change. Subsequently regeneration takes place, under favourable circumstances, and may be followed by nodular hyperplasia of liver cells, or the formation of tumour-like masses showing the characters of hepatomas and excessive proliferation of bile duct epithelium. The lesions are essentially similar to some of those produced by *p*-dimethylaminoazobenzene (butter yellow), and described by many workers, notably by Opie (1944).

In the absence of metastases it is not certain that the changes observed represent stages of an irreversible neoplastic process. There is little reason to doubt, however, that with survival over a longer period truly malignant characters may supervene.

The finding that liver tumours are produced in rats treated intermittently for more than 8 months with alkaloids of *Senecio jacobaea* raises interesting problems. The question how far the results described above bear on the problem of primary liver tumours in man suggests clinical inquiry about herbal treatment of the respective patients. However, the prevalence of this type of tumour among populations known to use concoctions of *Senecio* plants from childhood suggests that this practice may play a part in the aetiology of primary liver tumours.

*Senecio* plants are being still sold in Great Britain for treatment of colds, coughs, sciatica, pains in limbs and other disorders (Potter and Wren, 1941; Gerarde, 1597). The recommended dose of an infusion of one ounce of dried plant in a pint of water is a wineglassful. Assuming the alkaloid content of the dried plant to be of the order 0·06 per cent (Barger and Blackie, 1937), such an infusion would contain about 0·03 mg./ml., a concentration comparable with that used in our experiments (0·05–0·1 mg./ml.). In view of the chronic or recurrent nature of the disorders for which such treatment is recommended, its danger is obvious.

Recently, Hoch-Ligeti (1949) reported the production of liver tumours in 12 out of 21 rats killed after 7 months' feeding of 10 per cent. chilli (*Capsicum frutescens* and *C. annuum*) in a semi-synthetic deficient diet; this contained ardein, the protein of ground nuts. In a previous experiment of the same worker (1948), when the rats received 10 per cent chilli in a semi-synthetic diet, containing, however, an animal protein, casein, for 14 to 24 months, 7 out of 50 experimental rats developed liver changes resembling hepatoma and cholangioma, three of them malignant. In these experiments the type of diet seems to have had a contributory action. In our experiments the animals were fed the well-balanced rat cakes *ad libitum*. However, the weight of the rats was markedly influenced by the consumption of the alkaloids, and was below that of the controls.

That deficient diet of the type used by the Bantus is not sufficient to induce liver tumours has been shown by the experiments of Gillman (1944), and Gillman, Gillman, Mandelstam and Gilbert (1945). These workers fed newly weaned albino rats with maize-meal porridge (mealie-pap) and sour milk (the staple diet of the South African negro) for up to 15 months. Although severe liver injury was produced, no tumours developed.

### SUMMARY.

Liver tumours have been observed in 3 albino rats which survived more than 8 months of intermittent feeding with alkaloids of *Senecio jacobaea*. The possible bearing of these results on the aetiology of primary liver tumours frequent among the negro population of South Africa is discussed in the light of the indiscriminate use of *Senecio* plants for treatment of numerous disorders.

These experiments were begun in the animal house of the Materia Medica Department, where facilities were kindly placed at our disposal. We are greatly indebted to Professor C. M. Yonge, F.R.S., for the provision of alternative accommodation in the Zoology Department, which enabled the work to be continued when these facilities were withdrawn. Our thanks are due to Miss E. M. Ross and Miss E. B. Duff for excellent care of the animals, and the former for valuable technical assistance ; also to Mr. S. Breslin for photographic reproductions and histological sections. This work has been supported by a grant from the British Empire Cancer Campaign.

### REFERENCES.

ADAMS, R., AND LEONARD, N. J.—(1944), *J. Amer. chem. Soc.*, **66**, 257.
BARGER, G., AND BLACKIE, J. J.—(1937) *J. chem. Soc.*, p. 584.
CUSHNY, A. R.—(1911) *J. Pharmacol.*, **2**, 531.
*Idem* AND WATT, H. E.—(1920) *Lancet*, ii, 1089.
DAVIDSON, J.—(1935) *J. Path. Bact.*, **40**, 285.
GERARDE, J.—(1597) 'The Herball or Generall Historie of Plantes.' London, p. 219.
GILLMAN, J.—(1944) *Brit. med. J.*, i, 149.
*Idem*, GILLMAN, T., MANDELSTAM, J., AND GILBERT, C.—(1945) *Brit. J. exp. Path.*, **26**, 67.
HARRIS, P. N., ANDERSON, R. C., AND CHEN, K. K.—(1942) *J. Pharmacol.*, **75**, 78.
HENRY, T. A.—(1949) 'The Plant Alkaloids.' London (J. & A. Churchill), p. 601.
HOCH-LIGETI, C.—(1948) *Ann. Rep. Brit. Emp. Cancer Campgn.*, **26**, 118.—(1949) *Ibid.*, **27**, 138.
KENNAWAY, E. L.—(1944) *Cancer Res.*, **4**, 571.
LEONARD, N. J.—(1950) 'The Alkaloids,' **1**, 107–164 (Editors, Manske, R. H. F., and Holmes, H. L.). New York (Academic Press).
OPIE, E. L.—(1944) *J. exp. Med.*, **80**, 231.
POTTER, H., AND WREN, R. W.—(1941) 'Potter's Cyclopaedia of Botanical Drugs and Preparations.' London (Potter and Clarke, Ltd.), p. 290
ROSE, C. L., FINK, R. D., HARRIS, P. N., AND CHEN, K. K.—(1945), *J. Pharmacol.*, **83**, 265.
ROSENFELD, I., AND BEATH, O. A.—(1945) *Amer. J. clin. Path.*, **15**, 407.
WATT, J. M., AND BREYER-BRANDWIJK, M. G.—(1932) 'The Medicinal and Poisonous Plants of Southern Africa.' Edinburgh (E. & S. Livingstone), pp. 198–207.

---

### EXPLANATION OF PLATES.

FIG. 1.—Section 516 of liver from a rat which died during the first month of treatment with *S. jacobaea* alkaloids, showing necrotic and degenerative changes.

FIG. 2.—Section 511 of liver of a rat which died after 6½ months of treatment, showing besides degenerative-hyperplastic changes ; note cytoplasmic vacuolation, abnormal and variable nuclear patterns.

FIG. 3.—Section 569 of liver from a rat which died after 7 months' treatment showing features similar to Fig. 2.

FIG. 4.—Section 423 of liver from a rat sacrificed after 8 months of treatment showing hepatomatous growth (diffuse and focal regeneration).

FIG. 5, 6, 7.—Different areas of section 568 of liver tumour from a rat which died after 10 months' treatment.

FIG. 8.—Section 454 of liver tumour from a rat sacrificed after 11 months of treatment.

Cook, Duffy and Schoental.

Cook, Duffy and Schoental,